# Embryonic GABA_B_ Receptor Blockade Alters Cell Migration, Adult Hypothalamic Structure, and Anxiety- and Depression-Like Behaviors Sex Specifically in Mice

**DOI:** 10.1371/journal.pone.0106015

**Published:** 2014-08-27

**Authors:** Matthew S. Stratton, Michelle Staros, Tomaz Budefeld, Brian T. Searcy, Connor Nash, Chad Eitel, David Carbone, Robert J. Handa, Gregor Majdic, Stuart A. Tobet

**Affiliations:** 1 Department of Biomedical Sciences, Colorado State University, Fort Collins, Colorado, United States of America; 2 Center for Animal Genomics, Veterinary Faculty, University of Ljubljana, Ljubljana, Slovenia; 3 Department of Basic Medical Sciences, University of Arizona College of Medicine, Phoenix, Arizona, United States of America; 4 School of Biomedical Engineering, Colorado State University, Fort Collins, Colorado, United States of America; University of Otago, New Zealand

## Abstract

Neurons of the paraventricular nucleus of the hypothalamus (PVN) regulate the hypothalamic- pituitary-adrenal (HPA) axis and the autonomic nervous system. Females lacking functional GABA_B_ receptors because of a genetic disruption of the R1 subunit have altered cellular characteristics in and around the PVN at birth. The genetic disruption precluded appropriate assessments of physiology or behavior in adulthood. The current study was conducted to test the long term impact of a temporally restricting pharmacological blockade of the GABA_B_ receptor to a 7-day critical period (E11–E17) during embryonic development. Experiments tested the role of GABA_B_ receptor signaling in fetal development of the PVN and later adult capacities for adult stress related behaviors and physiology. In organotypic slices containing fetal PVN, there was a female specific, 52% increase in cell movement speeds with GABA_B_ receptor antagonist treatment that was consistent with a sex-dependent lateral displacement of cells *in vivo* following 7 days of fetal exposure to GABA_B_ receptor antagonist. Anxiety-like and depression-like behaviors, open-field activity, and HPA mediated responses to restraint stress were measured in adult offspring of mothers treated with GABA_B_ receptor antagonist. Embryonic exposure to GABA_B_ receptor antagonist resulted in reduced HPA axis activation following restraint stress and reduced depression-like behaviors. There was also increased anxiety-like behavior selectively in females and hyperactivity in males. A sex dependent response to disruptions of GABA_B_ receptor signaling was identified for PVN formation and key aspects of physiology and behavior. These changes correspond to sex specific prevalence in similar human disorders, namely anxiety disorders and hyperactivity.

## Introduction

The formation of functional circuits in the developing brain depends on the orchestrated regulation of cellular developmental processes including cell division, signal induced cell death, cell migration, specification, axon outgrowth and connectivity. These processes are dependent on a combination of genetic programming and responses to external cues [1]. In the hypothalamus, the paraventricular nucleus (PVN) is the final common neuronal pathway for regulation of the hypothalamic - pituitary - adrenal (HPA) axis, which provides a key hormonal component of stress responses [2]. The neurons of the PVN also help regulate the autonomic nervous system and thereby, cardiovascular functions [3]. Dysregulation of the HPA axis has been implicated in human anxiety and depression related disorders, both of which display significant sex dependence [4].

In the fetal mouse brain, the developing PVN is surrounded by cellular elements containing high levels of the GABA synthesizing enzymes, glutamic acid decarboxylase (GAD) 65 and 67 (also referred to as GAD2 and 1, respectively in humans), as well as GABA itself. At the same time, cells within the developing PVN contain abundant amounts of GABA receptors [5]. The receptor expression pattern provides a basis for the hypothesis that GABA directs key developmental steps in this brain region. Previous work showed that disruption of the gene encoding the GABA_B_ receptor led to altered protein expression and cell placement in and around the neonatal PVN [5], [6]. In addition, embryonic treatment with a GABA_A_ receptor antagonist (bicuculline) decreased the number of cells containing immunoreactive estrogen receptors (ER-alpha specifically) within the PVN, again in neonates [5]. The current study was designed to determine the role of GABA_B_ receptor signaling on the formation of the PVN and potential adult consequences of blocking GABA_B_ receptor signaling during embryonic development. The evidence for altered GABA signaling contributing to depression and anxiety disorders is growing (reviewed; [7]–[10]). There are decreased GABA concentrations in plasma [11], CSF and brain [12]–[14], and fewer GABA neurons in the orbitofrontal cortex of individuals with various forms of depression [15]. There are links for polymorphisms of the GAD 65 gene with anxiety behaviors in children [16] and of the GAD 67 gene with depression in women [17]. Also, GAD 67 is less abundant in the brains of patients with mood disorders relative to controls [18]. In animal models, disrupting GAD genes [19], [20], GABA_A_, or GABA_B_ receptors [21]–[23] all cause anxiety-like phenotypes and some have been reported to cause changes in depression-like behavior. In these cases, even though the given mutations and chemical imbalances are present throughout development, results have been interpreted from the perspective of GABA acting in adults to regulate neuronal excitability without investigation of developmental processes.

Understanding fetal antecedents to adult diseases may provide key tools for diagnosis and treatment of many debilitating conditions [24]. The current work tested the effect of GABA_B_ receptor blockade on acute cell movements in the embryonic PVN *ex vivo* and subsequent behavioral and physiological consequences of similar treatments that resulted in altered cell placements in and around the PVN. Pharmacological blockade was chosen for these studies to restrict the treatment to a critical fetal period and compare to prior work with a genetic disruption that is present throughout the lifespan.

## Materials and Methods

### Animals

**Figure 1:**
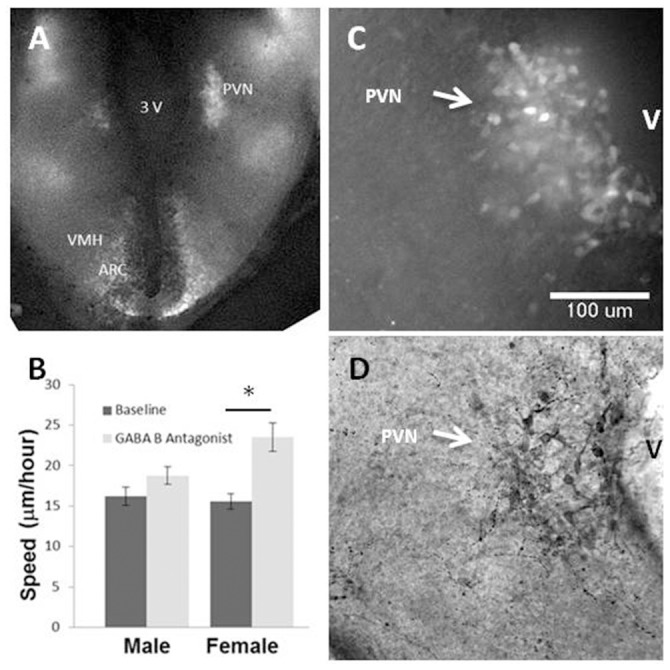
Effects of GABA_B_ receptor antagonists 2 Hydroxy Saclofen and CGP 55845 on cell movements in *ex vivo* brain slice cultures. GABA_B_ receptor antagonists caused cells in slices from female embryos, but not male embryos, to increase their speed of movement. Panel A shows a low magnification image of fluorescence in the hypothalamus of Thy1 YFP mice. Labeled neurons are also seen in the arcuate (ARC) and ventromedial nuclei (VMH). The data is summarized by sex and treatment in panel B, illustrating a significant effect of antagonists on rate of motion only for cells from slices taken from female embryos (statistically significant sex × treatment interaction, p<0.05). (C) Representative image of fluorescently labeled neurons in the developing PVN. A subset of slices was processed for CRH immunohistochemistry (D) to verify that video recordings were taken from developing PVN. (female n = 50 cells from 14 slices; male n = 34 cells from 16 slices).

B6.Cg-Tg (Thy1-YFP)16Jrs/J transgenic mice [25] were used for fluorescence video microscopy as described previously. The Thy-1 promoter drives YFP neuronal expression in the brain and allows for tracking of individual immature neurons in specific regions of the developing hypothalamus [26], including the PVN in the current study (e.g., figure 1A, C).

To block GABA_B_ receptor activation during PVN development, time mated pregnant dams (C57BL/6J) were injected (s.c.) with 0.1 mg/kg CGP 55845 [27] (Tocris Biosciences) or vehicle (1% DMSO, 0.25% EtOH, PBS) daily from E11 through E17. Five pregnant dams were treated with CGP 55845 and five pregnant dams were treated with vehicle. Offspring were weaned at postnatal day (P) 19, assigned random numbers and marked by ear punch. At 11 weeks of age animals started behavior testing and restraint stress HPA axis activation testing. At the conclusion of testing animals were perfused through the heart with 4% paraformaldehyde and immunohistochemical analyses were conducted.

### Ethics Statement

All experiments were carried out in accordance with the NIH Guide for the Care and Use of Laboratory Animals and approval of the Colorado State University Institutional Animal Care and Use Committee.

### Fluorescence Video Microscopy

Fetal mice were taken at embryonic day (E) 13, a developmental age when the majority of cells destined to become part of the PVN have had their last mitotic event but have not yet completed their migration. Live slice preparations followed previously published methods [26], [28]. Briefly, dissections were limited to a maximum of two hours to minimize cellular damage. Following each dissection, brains were embedded in 8% agarose and cut in a coronal plane at 250 µm in Krebs buffer using a vibrating microtome (Leica VT1000S). Slices that contained regions of the hypothalamus that included the PVN were chosen for video microscopy and were transferred to culture media (Hybernate-E; GIBCO-Invitrogen Corporation, Carlsbad, CA), with 2% pen-strep, and incubated at 36°C with 5% CO_2_ for 30 minutes. Following the 30-minute incubation period, each slice was plated onto glass bottom dishes pre-coated by the manufacturer with poly-d-lysine (MatTek; P35G-0-20-C) and coated with an additional 1∶1 dilution of Purecol (Advanced Biomatrix, San Diego, CA) and water. The slices were put back into the 36°C incubator for up to 30 minutes to promote adhesion. To prevent slice movement during video observation, 40 µL of a collagen solution (1 mL Purecol, 125 µL 10XMEM, 23 µL penicillin/streptomycin, and 33 µL 1 M sodium bicarbonate) was placed over each slice. The slices were then placed in the 36°C incubator for up to 1 hour to allow the Purcol to polymerize. 1 mL of Neurobasal media (10% L-glutamine, 2% B-27, 1.1% glucose, 2% pen-strep, 2% glutamine) was pipetted into each dish. The slices were maintained at 36°C and 5% CO_2_ until use for video microscopy - as early as the following morning and as late as 2 days post plating.

In preparation for video microscopy, slices were washed with warm Neurobasal media (GIBCO-Invitrogen), and placed on a heated stage maintained at 37°C with 5% CO_2_ with fresh Neurobasal medium. Data were collected using an inverted Nikon TE2000-U (Nikon USA, Melville, NY) microscope with a 20x plan Apo phase objective (0.75 na) or an upright Olympus BX61W (Olympus America, Center Valley, PA) microscope with an XLUM PLAN FLN-W 20x objective (1.0 na). Cells expressing yellow fluorescent protein (YFP) were imaged using Metamorph software (Molecular Devices Inc., Sunnyvale, CA). At least 1.5 hours of baseline video microscopy was taken before the addition of GABA_B_ receptor antagonists, 2-hydroxy saclofen ([29]; Sigma-Aldrich, St. Louis, MO) or CGP 55845 ([30]; Tocris Biosciences, Bristol, UK). Then an additional 1.5 hours of video was taken to compare baseline to treatments. Vehicle was used to serve as an additional control. E13 embryos have only 1 or 2, sections that contain developing PVN when sectioned at 250 µm.

### Immunohistochemistry

Immunohistochemistry was performed as previously reported [6]. Briefly, fixed tissue was embedded in 5% agarose and cut into coronal 50 µm thick sections using a vibrating microtome (Leica VT1000S). Alternating sections were collected in 0.05 M phosphate buffered saline (PBS), pH 7.5. Excess unreacted aldehyde was neutralized using a 30-minute incubation in 0.1 M glycine and 15 minutes in 0.5% sodium borohydride in PBS. After washing the tissue sections in PBS the sections were incubated in a PBS blocking solution containing 5% normal goat serum, 0.3% Triton-X 100 (Tx) and 1% hydrogen peroxide for 60 minutes at 4°C. Tissue was then incubated in primary antisera containing 1% bovine serum albumin (BSA) and 0.3%Tx over 2 nights at 4°C. Primary antisera were used at a 1∶10,000 dilution (rabbit anti-ERα; C1355, Upstate Biotechnology, Charlottesville, VA) or a 1∶5,000 dilution (rabbit anti-cFOS; sc-52, Santa Cruz Biotechnology, Santa Cruz, CA). Anti-rabbit secondary antiserum was diluted to 1∶2500 (Rabbit IgG-fab fragment; Jackson Immunoresearch, West Grove, PA). Sections were washed at room temperature in PBS containing 1% normal goat serum and 0.02% Tx then incubated at room temperature in secondary antisera buffer containing 1% normal goat serum and 0.32% Tx with a biotin conjugated anti-rabbit secondary diluted to 1∶2500 (Rabbit IgG-fab fragment; Jackson Immunoresearch, West Grove, PA). Sections were developed using a Vectastain ABC Elite kit (Vector Laboratories; Burlingame, CA) for 1 hour at room temperature and then exposed for 5 minutes to a solution containing 0.025% diaminobenzidine with 0.02% nickel ammonium sulfate and 0.02% H_2_O_2_ diluted in tris-buffered saline (pH 7.5).

### Radioimmunoassay (RIA)

Blood was collected via the tail vain at the start of restraint stress (placement in a 50 ml plastic conical tube) and after 30 minutes of restraint stress. Plasma corticosterone concentrations were measured by RIA. Plasma samples were diluted 1∶25 in 0.01 M PBS, and corticosterone binding globulin was denatured by incubating the samples at 65°C for one hour. All samples and standards were then incubated overnight at 4°C in the presence of antiserum (MP Biomedicals, Solon OH) and [3H] corticosterone (Perkin- Elmer, Boston, MA) in 0.1% gelatin dissolved in 0.01 M PBS. Unbound corticosterone was removed by adding dextran-coated charcoal, which was then separated by centrifugation. Bound corticosterone was decanted into new vials, mixed with scintillation fluid, and counted using a Beckman Coulter LS6500 Multipurpose Scintillation Counter (Brea, CA). Plasma corticosterone concentrations were determined by comparison to a standard curve (5–700 ng/ml). The intra-assay variance was 3.26%, and was determined by an internal control that was measured at regular intervals throughout the assay.

### Analysis

Images of the PVN were carefully matched by an investigator blinded to treatment condition to ensure that the same section (relative to rostral-caudal location) was used in analyses for each animal. Images of sections containing immunoreactive ERα were normalized for optimal contrast in Adobe Photoshop (version CS for Macintosh) and immunoreactive area above threshold (not densitometry) was quantified in IP Lab Imaging software (Scanalytics Inc., part of BD Biosciences, Rockville, MD) as reported previously [5]. Eight 100 µm (width) × 500 µm (height) columns were placed over the images starting 100 µm lateral from the edge of the ventricle (to eliminate impact of an immunoreactive edge artifact) and 200 µm above the top of the ventricle. Cells containing immunoreactive ERα were selected for in vivo cell placement analysis due to their sensitivity to displacement in previous studies and because their clearly identifiable cell bodies/nuclei were amenable to quantification. Other immunoreactive components of the PVN (i.e. vasopressin or corticotropin releasing factor) were not selected for cell placement analysis due complications of immunoreactive content in axons and dendrites relative to the placement of cell bodies. For FOS analysis, an investigator manually counted FOS immunoreactive cell nuclei in the PVN (both left and right sides) from two sections per animal in the mid-region of the PVN.

For analysis of the distribution of ERα immunoreactive cells, significance was determined by three way ANOVA analyzing Sex × Treatment × Location (columns as a repeated measure) with SPSS software (SPSS Inc., Chicago, IL). See results section for additional detail on posthoc analyses.

For all behavior testing and HPA axis activation assessment, sex differences in response to CGP 55845 were determined by analyzing the Sex × Treatment interaction in two-way ANOVAs along with examining the main effects of sex and treatment. Once an overall significance was established via ANOVA, posthoc group differences were based on non-overlapping 95% confidence intervals. All data are expressed as mean±SEM.

### Behavior Testing

Behavior testing (details in supplemental material) started when mice were approximately 11 weeks of age. Females were tested while in estrus as determined by primarily cornified epithelial cells in the vaginal cytology. Animals were handled for two weeks prior to the start of testing and for two days before each individual test. The testing order was Elevated Plus Maze, Open Field, Tail Suspension, Forced Swim (Figure S1 in file S1), Sucrose Preference, and Restraint Stress for each animal. Except for the sucrose preference test, the testing order was least stressful to most stressful. Sucrose preference test was assessed after forced swim testing because animals must be housed individually to measure individual fluid intake. The intention was to avoid confounding effects of social isolation and/or re-socialization for the rest of the behavior tests. Restraint stress was assessed last so that FOS activation could be quantified via immunohistochemistry at an acute time point following restraint stress. Tests were conducted during the same 4-hour window each day (11∶00 am–3∶00 pm) in the light phase of the animal’s daily cycle. Tests were spaced at least 4 days apart for each animal. During each testing day the order of animal testing (i.e. sex and treatment) was randomized. Analysis was blinded through a random animal numbering system. Thirty-six animals were behavior tested and each group consisted of offspring from at least three different nulliparous dams. Specific numbers of animals that were included in each analysis are reported in the appropriate figure legends.

## Results

### *Ex Vivo* cell movement with GABA_B_ receptor blockade

To determine the effect of GABA_B_ receptor antagonists on cell movement in the developing PVN, mouse embryos were harvested on E13 and organotypic slices containing fluorescently labeled neurons in the developing PVN (Figure 1A, 1C) were maintained in vitro. Blockade of GABA_B_ receptors in these brain slices resulted in increased rates of cell movement in the developing PVN in a sex specific manner (Figure 1B). Cells in organotypic slices from female but not male embryos increased their speed (µm/h) of migration by 52% following GABA_B_ receptor antagonist treatment compared to baseline movement (female baseline = 15.6±0.93 female antagonist = 23.5±1.7, Sex × Treatment ANOVA F(1,82) = 5.56, p<0.05) (male baseline = 16.21±1.16, male antagonist = 18.7±1.08) (Figure 1B). Immunoreactive vasopressin and corticotropin releasing hormone (CRH) provided posthoc evidence that sections chosen for video microscopy contained authentic PVN (e.g., Figure 1D). The speed of cell movements were affected similarly regardless of which GABA_B_ receptor antagonist was used (no significant difference between 1 µM CGP55845 and 20 µM 2-hydroxy saclofen). Vehicle treatment did not alter cell movement speeds (Supplemental table S1 in file S1).

### *In Vivo* cell placement following fetal GABA_B_ receptor blockade

Cells containing immunoreactive ERα were present in the PVN in a pattern extending laterally from the third ventricle to juxtaparaventricular and perifornical areas [31], [32]. A pilot experiment examined the placement of cells containing immunoreactive ERα at birth following embryonic treatment with CGP 55845 (data not shown). The results provided a positive basis to continue raising the cohort of mice that were tested for behavior and ultimately analyzed for immunoreactive ERα in adulthood (see below).

**Figure 2:**
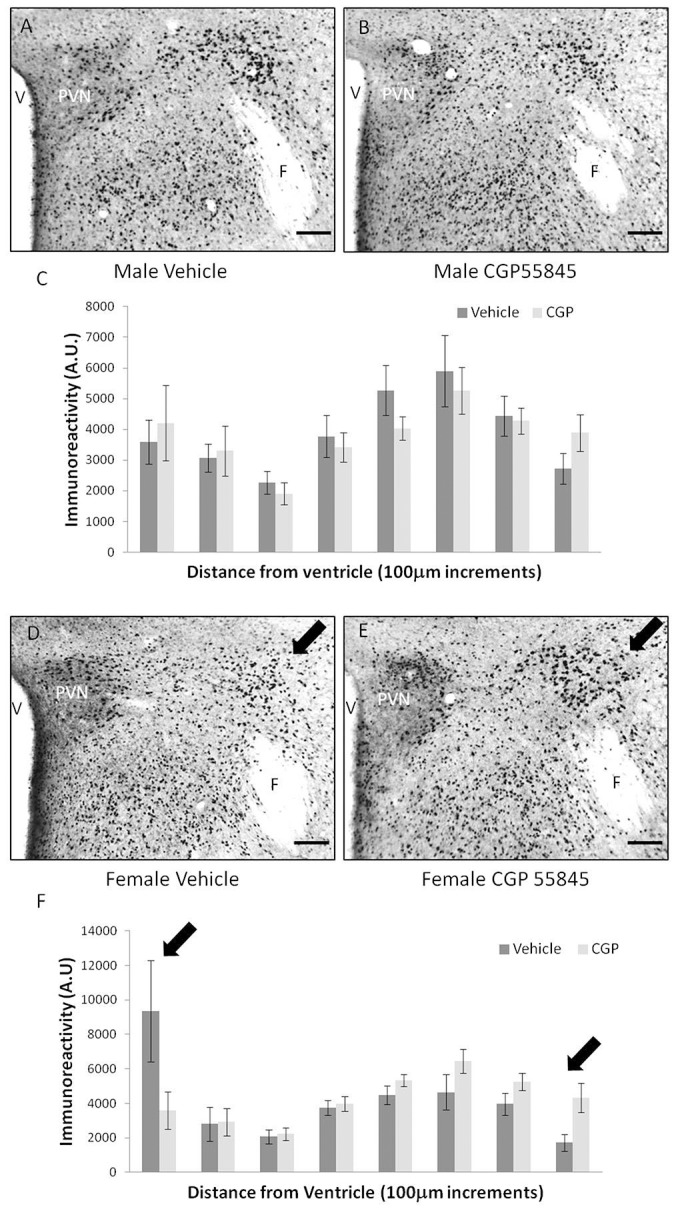
Distribution of cells containing immunoreactive ERα in the region of the PVN. When mothers were treated with CGP 55845, male offspring (Panels A–C) had no difference in the position of immunoreactive ERα containing cells (B) relative to vehicle treated controls (A). The distribution of immunoreactive cells relative to distance from the third ventricle is shown quantitatively in panel C. In female offspring of mothers treated with vehicle (Panels D,F) a large number of immunoreactive cells were concentrated close to the third ventricle (V). By contrast, when mothers were treated with CGP 55845, female offspring (Panel E,F) had fewer cells noted in medal positions and more cells were located in positions lateral to the paraventricular nucleus (PVN) and closer to the perifornical area (black arrows). Post hoc analysis revealed that CGP 55845 selectively shifted this immunoreactivity in females (medial to lateral indicated by black arrows panel F) (p<0.05). F in panels A, B, D, E = fornix, scale bars represent 200 µm. (n = 6 vehicle male, n = 7 CGP 55845 male, n = 6 vehicle female, n = 7 CGP 55845 female).

Fetal treatment with the GABA_B_ receptor antagonist CGP 55845 reproduced in adults the phenotype seen previously in the neonatal GABA_B_ R1 subunit knockout mouse [5]. In female offspring from mothers treated with CGP 55845 there was a lateral shift in the distribution of cells containing immunoreactive ERα toward juxtaparaventricular and perifornical areas (Figure 2D–F). There was no difference in the positions of cells in males from the same litters (figure 2A–C). In the most medial column quantified, females treated with CGP 55845 had 71% less immunoreactivity compared to controls while in the two most lateral columns quantified, females treated with CGP 55845 had 60% more immunoreactivity compared to vehicle treated animals (Sex × Treatment × Location as a repeated measure; F(7,154) = 3.20, p<0.05). To determine to what extent the significant 3-way interaction was due selectively to females versus males, orthogonal contrasts were run for each sex individually in a Treatment × Location as repeated measure posthoc analysis. There was statistical significance in females (F(7,154) = 5.28, p<0.01), but not males (F(7,154) = 0.46, p = 0.86).

### Increased anxiety-like behavior in female offspring of mothers treated with GABA_B_ receptor antagonist

**Figure 3:**
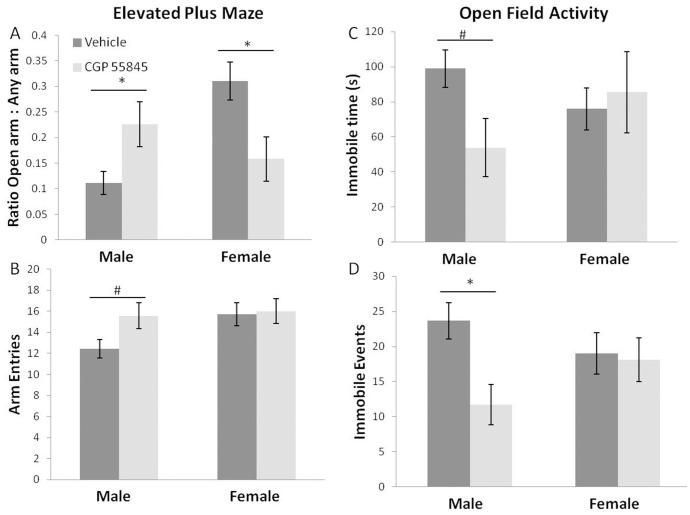
Anxiety- and hyperactivity-like behaviors: Elevated Plus Maze and activity in an Open Field. Panel A depicts the open arm ratio of the Elevated Plus Maze test (time in open arms divided by time spent in any of the four arms), while panel B depicts the total number of arm entries for the elevated plus maze test. Female offspring of mothers treated with CGP 55845 spent less time in the open arm compared to vehicle controls. Male offspring of mothers treated with CGP 55845 showed increased open arm ratio and an increased trend in number of arm entries (n = 9 vehicle male, n = 7 CGP 55845 male, n = 11 vehicle female, n = 9 CGP 55845 female). As measures of activity in an ‘open-field’, time spent immobile (C) and number of immobile events (D) were quantified. Male offspring from mothers treated with CGP 55845 during pregnancy displayed a hyperactivity-like phenotype that was most obvious for the number of immobile events (D). Males also showed decreased latency to immobility with embryonic exposure to the GABA_B_ receptor antagonist (see text) and a trend toward decreased time spent immobile. There were no obvious treatment effects on the activity of females (n = 9 vehicle male, n = 7 CGP 55845 male, n = 11 vehicle female, n = 9 CGP 55845 female). (*indicates non-overlapping 95% confidence interval following significant treatment effect or significant Sex × Treatment interaction).

Adult offspring of dams treated with CGP 55845 or vehicle were subjected to the elevated plus maze test to assess anxiety-like behavior [33]. Female offspring of CGP 55845 treated mothers showed significantly increased anxiety-like behavior. This was indicated by decreased time in the open arms of an elevated plus maze (ratio of time spent in open arm divided by time spent in any arm; Figure 3A, vehicle = 0.31±0.04, CGP 55845 = 0.158±0.04; Figure 3A). Male offspring of fetal CGP 55845 treated mothers showed increased time in the open arms (Figure 3A, vehicle = 0.11±0.02; CGP 55845 = 0.23±0.04), which may be indicative of decreased anxiety-like behavior. The sex difference in the effect of exposure to fetal CGP exposure was established by a statistically significant Sex × Treatment interaction (F(1,31) = 12.50, p<0.05).

To interpret a finding of increased time in open arms as less “anxiety-like” behavior one must control for total activity of the animals. The increased time in the open arms in male offspring of CGP treated mothers was accompanied by a trend toward an increased total number of arm entries. This suggested increased activity (entries vehicle = 12.4±0.86; CGP 55845 = 15.6±1.2), although there was no significant main effect of treatment or Sex × Treatment interaction. Male open arm ratios and male total arm entries were significantly correlated (R = 0.688, p<0.005). Overall, females were more active than males in the EPM as indicated by more arm entries (female vehicle = 16.0±1.2; male vehicle = 12.4±0.86; F(1,31) = 4.4, p<0.05).

### Increased activity in adult male offspring of mothers treated with GABA_B_ receptor antagonist

The apparent male hyperactivity indicated by arm entries in the elevated plus maze with embryonic GABA_B_ receptor blockade was confirmed by analyzing animal mobility in an open field (Figure 3C, D). Total time spent mobile, latency to immobility (Figure S2 in file S1) and number of immobile episodes were quantified using Anymaze software (Stoelting Co.). There was a 45% decrease in time spent immobile in male offspring of CGP 55845 treated mothers relative to controls (Figure 3C, immobile time male vehicle = 98.9±10.7; male CGP 55845 = 53.9±16.6) without significant main effects of treatment or sex due to variability within the females. However, when males were analyzed alone in a two tailed T test, statistical significance was attained when comparing vehicle and CGP 55845 treatment (p<0.05). Also, male offspring from mothers treated with CGP 55845 showed significantly increased latency to immobility (latency in seconds: male vehicle = 45.1±12.8; male CGP 55845 = 125.9±29.2; Sex × Treatment ANOVA F(1,32) = 7.57, p<0.05) and significantly decreased immobile episodes relative to controls (Figure 3D, male vehicle = 23.7±2.5, male CGP 55845 = 11.7±2.8; Sex × Treatment ANOVA F(1,32) = 12.4, p<0.05). This suggests that fetal GABA_B_ receptor blockade resulted in the male offspring growing up to be hyperactive. There was no indication of an effect of fetal CGP treatment on female activity. As detailed in the supplemental material and methods section, the dimensions of the open box used for activity assessment was smaller than that needed for an appropriate anxiety-like behavior assessment by analysis of time spent on the box’s interior vs time spent on the perimeter. Consequently, anxiety-like behavior was not assessed in this open field test.

### Less depression-like behavior adult offspring of mothers treated with GABA_B_ receptor antagonist

**Figure 4:**
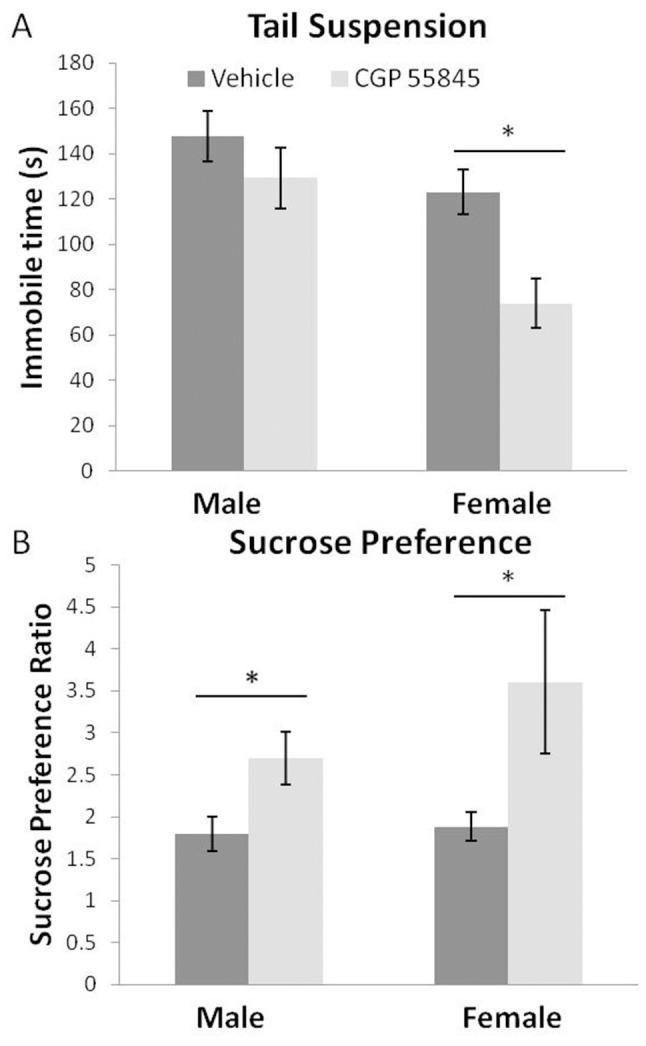
Measures of depression-like behavior: tail suspension and sucrose preference tests. Panel A shows that CGP 55845 treatment during pregnancy caused decreased immobility in the tail suspension test. However, only in females did the 95% confidence intervals not overlap (n = 6 vehicle male, n = 7 CGP 55845 male, n = 9 vehicle female, n = 9 CGP 55845 female). Panel B shows that male and female offspring exposed to fetal CGP 55845 had increased sucrose preference (n = 9 vehicle male, n = 7 CGP 55845 male, n = 11 vehicle female, n = 9 CGP 55845 female). (*indicates non overlapping 95% confidence intervals following significant main effect of treatment).

To assess the impact of developmental inhibition of GABA_B_ receptors on adult depression-like behavior, a tail suspension test was used. This test quantifies an animal’s “efforts” to fight/struggle out of what for most is an inescapable situation [33]. Mice are suspended by their tail for a period of five minutes and the time spent struggling and time spent motionless is counted. Male and female offspring of mothers treated with CGP 55845 displayed less depression-like behavior as indicated by shorter immobile times and increased struggling (Figure 4A). In females, time spent immobile was 66% less (female vehicle = 123.0±9.7; female CGP 55845 = 73.9±10.9) with fetal exposure to CGP 55845 while in males immobile time was 14% less (male vehicle = 147.7±11.2; male CGP 55845 = 129.2±13.4). There was a significant main effect of fetal treatment (F(1,27) = 8.67, p<0.05) and a significant main effect of sex (F(1,27) = 12.17, p<0.05). The Sex × Treatment interaction was not statistically significant, suggesting that male and female offspring were similarly affected by fetal treatment with CGP 55845. Nonetheless, the effect in females was almost 5 times larger than the effect in males and male 95% confidence intervals did overlap. Three males and two females “climbed their tails” and were excluded from this analysis on that basis. Interestingly, tail climbing was found only in the vehicle treated group.

### Less anhedonic/more hedonic-like behavior in adult offspring of mothers treated with GABA_B_ receptor antagonist

Depression- and anhedonic-like behavior was assessed using a sucrose preference test (Figure 4B). This test measures an animal’s pleasure seeking behavior by measuring its consumption of plain water compared to water sweetened with sucrose. Typically, when the constellation of depression-like behavior is increased, animals show no or reduced preference for sucrose over plain water which is interpreted as animals not going out of their way to seek pleasure [33]. Fetal exposure of males and females to CGP 55845 resulted in increased preference for 1% sucrose solution versus water (Treatment effect F(1,31) = 6.9, p<0.05, with no Treatment × Sex interaction). The ratio of sucrose consumption to water consumption increased 70% from 1.84 for offspring of vehicle treated mothers to 3.18 for offspring of CGP 55845 treated mothers.

### Decreased adult HPA axis activation in adult offspring of mothers treated with GABA_B_ receptor antagonist

**Figure 5:**
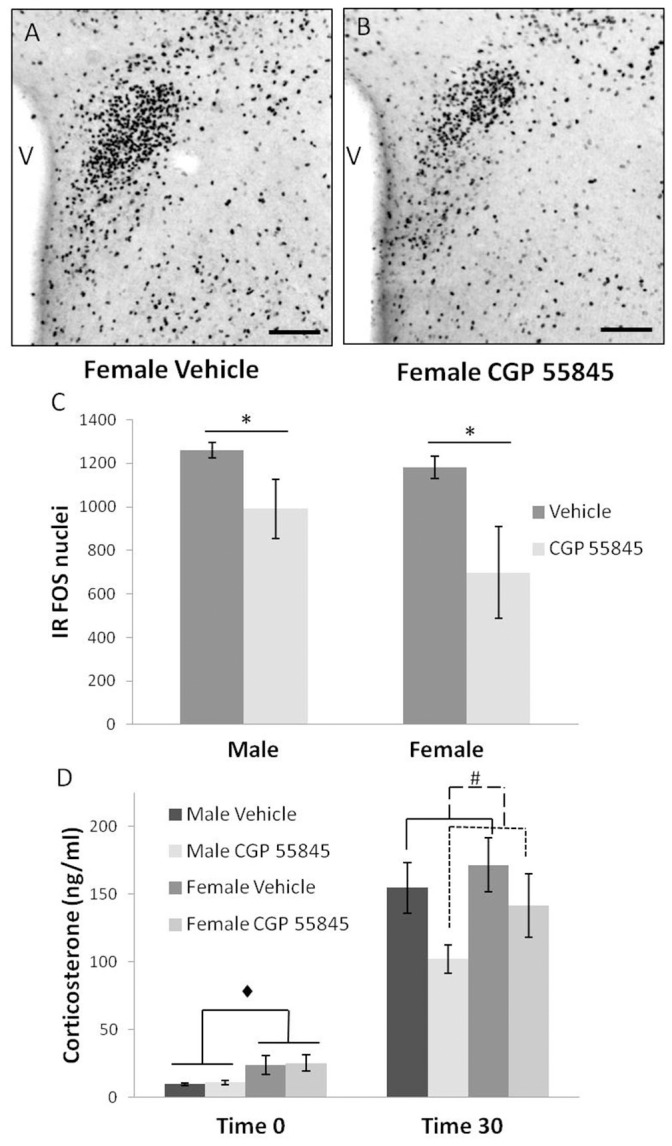
HPA axis output following acute stress. Immunoreactive Fos in the PVN one hour after release from restraint, vehicle treated (A) and CGP 55845 treated (B) female mice. Adult offspring of mothers treated with CGP 55845 during pregnancy had less immunoreactive Fos in the PVN one hour after release from restraint stress compared to controls in both males and females (C; n = 4 vehicle male, n = 4 CGP 55845 male, n = 4 vehicle female, n = 4 CGP 55845 female). At baseline, females had increased plasma corticosterone relative to males. After 30 minutes of restraint stress, offspring from mothers treated with CGP 55845 had blunted stress responses indicated by a smaller increase in corticosterone concentration relative to vehicle treated controls (D) (*indicates non overlapping 95% confidence intervals following significant main effect of treatment; ♦ indicates significant main effect of sex; # indicates significant main effect of treatment; V = 3rd ventricle, scale bars represent 200 µm).

The neuronal responses in the HPA axis to stress were assessed using short-term physical restraint. One hour after release from restraint stress, neuronal activation in the PVN as indicated by immunoreactive Fos was markedly attenuated (31%) in offspring of mothers that were treated with GABA_B_ receptor antagonist relative to vehicle controls (Figure 5C; main effect of treatment F(1,12) = 8.5, p<0.05). This occurred similarly in males and females as there was no statistically significant Treatment × Sex interaction.

Plasma corticosterone was measured after restraint stress to assess neuroendocrine output of the HPA axis (Figure 5D). At the start of restraint stress (time 0), female corticosterone values were significantly higher than in males (male = 10.3±3.5, female = 24.6±3.4) based on the main effect of sex (F(1,29) = 8.46, p<0.05) without a Sex × Treatment interaction. After 30 minutes of restraint stress, offspring of dams treated with CGP 55845 displayed a blunted stress response as indicated by decreased plasma corticosterone levels relative to vehicle treated controls (vehicle = 163.0±13.1, CGP 55845 = 121.9±15.0). There was a significant main effect of treatment (F(1,31) = 4.3, p<0.05) without a Sex × Treatment interaction.

## Discussion

In the current study, treatment of pregnant mice with a GABA_B_ receptor antagonist for a restricted period resulted in offspring that had altered cell positions within and around the PVN. The same animals displayed a general pattern of increased anxiety-like behavior in females and increased hyperactivity in males along with decreased stress response and decreased depression-like behavior in both sexes (although tending toward greater effects in females). Genetic and environmental influences combine in the developing fetus to contribute to an individual’s ability to respond to stressors as an adult and the likelihood of developing pathology as a result of stress [24]. The results of the current study suggest that 1) GABA can act through GABA_B_ receptors to impact cell migration in the PVN of females selectively, and 2) that time-restricted maternal treatment with GABA_B_ receptor antagonist can alter the behaviors of adult offspring in ways that are directly related to animal models for human psychiatric disorders.

Direct application of GABA_B_ receptor antagonists caused neurons in the PVNs of organotypic slices from females ex vivo to increase their speed of movement. An *in vivo* correlate was seen as a lateral shift in the location of cells containing immunoreactive ERα from within the PVN to the perifornical area in adult female offspring of mothers treated with GABA_B_ receptor antagonist. The shift following fetal CGP treatment created a neuroanatomical phenocopy of a GABA_B_ R1 subunit knockout mouse in terms of changing the distribution of cells containing immunoreactive ERα in the PVN and juxtaparaventricular and perifornical areas [5]. The in vitro results are consistent with the in vivo results being a function of direct GABA_B_ antagonism in the fetus as opposed to an influence of GABA_B_ antagonism in the mother that then secondarily impacts the fetus. Taken together this provides additional evidence consistent with a hypothesis that GABA restricts cell migration for some neurons so that they remain within the PVN during fetal development. It is also an interesting complementary finding to studies in which prenatal stress has been shown to alter the migration of GABAergic neurons in the developing cerebral cortex [34]. Thus GABA may influence migration of other cells on the one hand in some places (e.g., PVN this study; [35], [36]) and GABAergic cells may be influenced by other fetal factors in other places (e.g., cerebral cortex, [34], [37]).

The perifornical region is part of the hypothalamic area controlling emotional responses (HACER) in the primate [38] and is involved in the regulation of cardiovascular responses to emotions [39]. This region has also been implicated in aggressive behaviors and autonomic cardiovascular responses [40], [41]. Estradiol can enhance the sensitivity of PVN CRH neurons in the HPA response to stress [42]. The lateral population of cells containing immunoreactive ERα is comprised of approximately 70% GABAergic neurons with projections to the PVN in the adult rat. Estradiol can block glucocorticoid dependent negative feedback on the PVN via ERα signaling in the perifornical region [43]. This lateral population of ERα containing cells may be part of the limbic inputs that exert an inhibitory tone on the PVN [44]. Changing the position or levels of ERα could change the ability of estrogens to modulate the HPA axis.

Why the developing PVN appears to be more dependent on GABA_B_ signaling in females than in males remains an open question. The simplest explanation would be that there are sex differences in the GABA concentration seen by the developing PVN or in the distribution of GABA_B_ receptors in the developing PVN. A preliminary survey of GABA_B_ R1 subunit immunoreactivity in embryonic mice suggested no difference in GABA_B_ R1 protein expression between the sexes in the PVN (Stratton and Tobet, unpublished observations). While sex differences in concentrations of GABA have been reported, the studies have either been in adult or early postnatal animals, and PVN was contained in tissue blocks that also contained other hypothalamic nuclei [45], [46].

Adult offspring of mothers treated with CGP 55845 displayed more anxiety-like behavior and less depressive-like behaviors if they were female. In male offspring from the same litters, there was a hyperactivity-like phenotype and less depressive-like behavior. These findings suggest that the same insult (maternal exposure to GABA_B_ receptor antagonist) may result in the phenotype of a female predominant disorder in females (anxiety) and the phenotype of a male predominant disorder in males (hyperactivity). Previous studies using GABA_B_ receptor knockout mice that could be raised to adulthood also reported increased anxiety and antidepressant-like behavior, but without any sex dependence [47]. Importantly, these studies taken together indicate that the behavioral phenotypes seen in mice lacking functional GABA_B_ receptors (throughout the life span) may be explained at least in part by brain patterning effects of GABA during embryonic development.

Cells of the PVN provide the ultimate output regulation for the HPA axis modulating the hormonal response to stress. Anxiety- and depressive-like behaviors in animal models can be manifested as a result of manipulating the HPA axis and increasing circulating levels of CRH [48]. The importance of the PVN and the HPA axis as it relates to anxiety-related disorders has also been suggested in human studies [49]–[51]. In the current study, maternal treatment with a GABA_B_ receptor antagonist resulted in offspring with altered cytoarchitecture in the region of the PVN and this altered cytoarchitecture correlated with altered anxiety- and depression-like behaviors. Unfortunately, a direct cause and effect relationship cannot be drawn as the entire developing embryo was exposed to the CGP 55845 compound. Many brain regions contribute to anxiety- and depression-like behaviors [52] and it was not feasible to assay each of them to determine if CGP 55845 administration altered their cellular or chemoarchitecture.

Despite the blunted stress response in both sexes, females showed increased anxiety-like behavior in the elevated plus maze. This apparent disparity may be related to the timing and relative stressful nature of the tests. For instance the stress response was measured with corticosterone concentrations after 30 minutes of stress (similar longer term measurement for Fos) while the elevated plus maze was conducted over 5 minutes and did not involve an acute stressor other than minor handling and the presence of open and elevated platforms. This highlights the complexities of the ontogeny of diseases like anxiety and depression and the shortcomings of current rodent behavior models in capturing realistic correlates of human disease. Of course other brain regions also likely impact rodent performance in elevate plus mazes [53]. Nonetheless, the increased anxiety-like behavior and decreased depression-like behavior replicated phenotypes seen in GABA_B_ receptor knockout mice [8]. The importance lies in the suggestion that the loss of GABA_B_ signaling may be most critical during a restricted fetal period rather than throughout life.

In summary, the results of the current study are consistent with the hypothesis that GABA acts through GABA_B_ receptors to direct cell migration in the developing female PVN and that altered GABA_B_ receptor signaling during development has life long, and possibly sex specific consequences for physiology and behavior [54]. Identifying and understanding fetal antecedents to adult psychiatric disorders will greatly enhance the ability to develop prevention and treatment strategies specific to sex, genotype and known environmental conditions (e.g., fetal and maternal stress, infection, medications; [55]).

## Supporting Information

File S1 **Contains detailed behavior testing methods, one supplemental table, and two supplemental figures.**
(DOCX)
