# Supplementary material for: Embryonic GABAB Receptor Blockade Alters Cell Migration, Adult Hypothalamic Structure, and Anxiety- and Depression-Like Behaviors Sex Specifically in Mice
Source: PLoS One. 2014 Aug 27;9(8):e106015. doi: 10.1371/journal.pone.0106015 (PMC4146593; doi:10.1371/journal.pone.0106015)
Supplement: File S1 — Contains detailed behavior testing methods, one supplemental table, and two supplemental figures. (DOCX) [file pone.0106015.s001.docx]

**Supplemental Material:**

This supplement contains detailed methods for behavior testing, one table, two figures with legends, and supplemental references.

**Detailed Description of Behavior Testing Methods:**

Elevated Plus Maze:

To determine if animals treated embryonically with CGP 55845 displayed altered anxiety-like behavior, the elevated plus maze test was used as previously described [sup ref 1]. The dimensions of the apparatus were; lane width 5 cm, arm length 35 cm, wall height 15 cm, leg height 40 cm (Stoelting, Wood Dale, Illinois). Animals were placed at the center of the EPM facing the open arm. The testing period was 5 minutes. Arm entries and durations were quantified.

Open Field:

The open field test has been previously used as a measure of anxiety-like behavior and general activity [sup ref 2]. The open field test in this experiment was conducted in a clear plastic storage bin with base dimensions of 30 cm x 37.5 cm. Endpoints were quantified indicating general activity. The testing period was 10 minutes. For analysis of the open field test the Anymaze (Stoelting, Wood Dale, Illinois) software program was used to measure mobility, immobility, and latency to immobility.

Tail Suspension:

The tail suspension test was used to measure despair-like behavior and was conducted as described previously [sup ref 3]. Briefly, mice were suspended by their tails, which were attached with adhesive tape (~ 1 cm from tip of the tail) to a horizontal rod. The testing period was 5 minutes. Times spent struggling or immobile were quantified. In several instances mice were able to climb their tails and were not counted in this analysis.

Forced swim:

As another measure of despair-like behavior, mice were tested in a version of the forced swim test modified for testing mice [sup ref 4]. Animals were placed in the cylinder for a pretest exposure for 6 min the day prior to testing and then monitored during another 6 min test on the actual testing day. The time spent swimming and immobile were quantified during video playback using the Stopwatch+ software program by an investigator blinded to sex and treatment. Females were subjected to the pretest exposure during proestrus and tested the following day while in estrus as confirmed by vaginal cytology.

Sucrose Preference:

To assess the impact of embryonic CGP 55845 treatment on anhedonic-like behavior, the sucrose preference test was conducted as previously described [sup refs 5,6] with minor modifications. Briefly, mice were moved to individual static caging to allow for the presence of two drinking bottles. Animals were allowed to acclimate to drinking from two bottles for two days (both bottles water). Then animals were acclimated to drinking 1% sucrose (w/v) for two days (both bottles 1% sucrose). On the 5th day animals were given a choice of water or 1% sucrose. Bottles were changed each day at the same time of day and weighed before being put in the cage and after removal (measured 24 hours of consumption). This was repeated three times to acquire four days of consumption preference data (one bottle water, one bottle 1% Sucrose). The side that contained sucrose was randomized on the first day and alternated each subsequent day.

**Supplemental Table S1**. Gross characterization of cell responsiveness to GABA_B_ receptor antagonist and vehicle treatment in organotypic slice.

|  | % cells with increased rate of movement | % cells with decreased rate of movement | % cells with no change in rate of movement |
| --- | --- | --- | --- |
| Male (antagonist) | 39 | 18 | 43 |
| Female  (antagonist) | 61 | 8 | 31 |
| Male (vehicle) | 25 | 17 | 58 |
| Female (vehicle) | 18 | 27 | 55 |


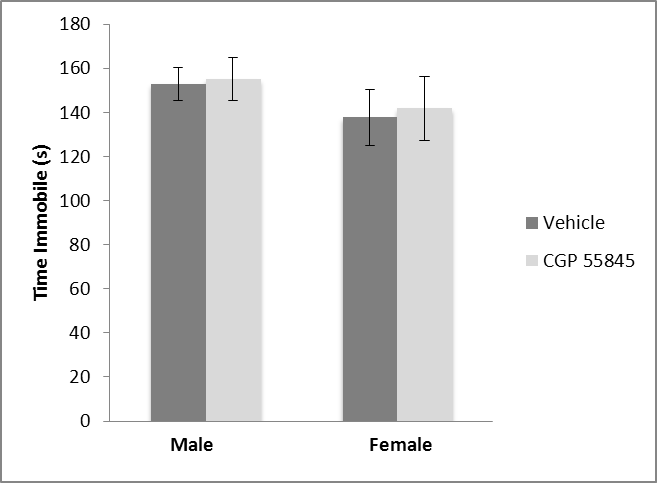


**Supplemental Figure S1**. No change in forced swim test behavior following fetal exposure to CGP 55845. Mice tested were offspring from pregnant mice treated with vehicle or CGP 55845 that were subjected to a forced swim test as adults. There were no significant differences noted in forced swim test behavior related to sex or treatment.


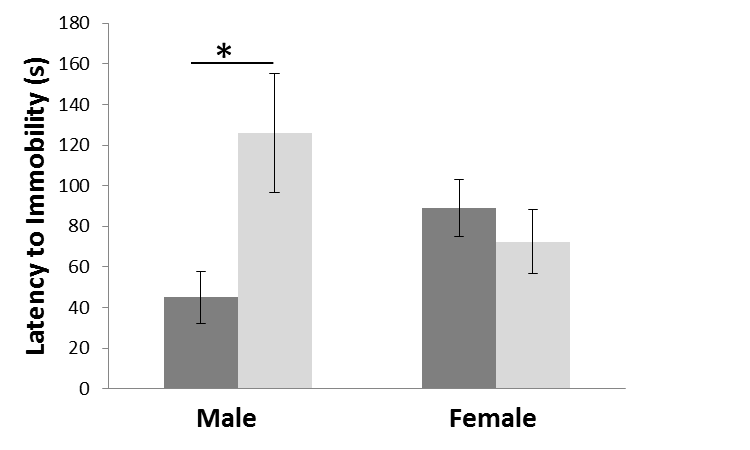


**Supplemental Figure S2**. Male mice from mothers treated with CGP 55845 displayed increased time to first immobile episode (latency to immobility). This is part of the general pattern of increased activity in males treated with CGP55845 in utero. (* indicates non overlapping 95% confidence intervals following significant sex X treatment interaction)

**Supplemental References:**

1. Weiser MJ, Wu TJ, Handa RJ (2009) Estrogen receptor-beta agonist diarylpropionitrile: biological activities of R- and S-enantiomers on behavior and hormonal response to stress. Endocrinology.150(4):1817-25.

2. Oyola MG, Portillo W, Reyna A, Foradori CD, Kudwa A, et al. (2012) Anxiolytic effects and neuroanatomical targets of estrogen receptor-beta (ERbeta) activation by a selective ERbeta agonist in female mice. Endocrinology 153:837-846.

3. Goel N, Bale TL (2008) Organizational and activational effects of testosterone on masculinization of female physiological and behavioral stress responses. Endocrinology 149:6399-6405

4. Bale TL, Vale WW (2003) Increased depression-like behaviors in corticotropin-releasing factor receptor-2-deficient mice: sexually dichotomous responses. J Neurosci 23:5295-301.

5. Mueller BR, Bale TL (2008) Sex-specific programming of offspring emotionality after stress early in pregnancy. J Neurosci 28:9055-65.

6. Poulter MO, Du L, Zhurov V, Merali Z, Anisman H (2010) Plasticity of the GABA(A) receptor subunit cassette in response to stressors in reactive versus resilient mice. Neuroscience 165:1039-1051.
